# Frailty Syndromes in Persons With Cerebrovascular Disease: A Systematic Review and Meta-Analysis

**DOI:** 10.3389/fneur.2019.01255

**Published:** 2019-11-29

**Authors:** Katie Palmer, Davide L. Vetrano, Luca Padua, Valeria Romano, Chiara Rivoiro, Bibiana Scelfo, Alessandra Marengoni, Roberto Bernabei, Graziano Onder

**Affiliations:** ^1^Department of Geriatrics, Centro Medicina dell'Invecchiamento, Università Cattolica del Sacro Cuore, Rome, Italy; ^2^Aging Research Center, Karolinska Institutet and Stockholm University, Stockholm, Sweden; ^3^Health Technology Assessment Department of the Institute for Economic and Social Research of Regione Piemonte, Turin, Italy; ^4^Department of Clinical and Experimental Sciences, University of Brescia, Lombardy, Italy; ^5^Department of Cardiovascular, Metabolic and Aging Diseases, Istituto Superiore di Sanità, Rome, Italy

**Keywords:** frail, cerebrovascular disease, stroke, vulnerable, aging, prefrail, geriatric, chronic disease

## Abstract

**Background:** Frailty can change the prognosis and treatment approach of chronic diseases. Among others, frailty has been associated with cerebrovascular diseases such as stroke. However, the extent to which the two conditions are related is unclear, and no systematic review of the literature has been conducted.

**Objectives:** To conduct a systematic review and meta-analysis assessing the association of cerebrovascular diseases and frailty, as well as prefrailty, in observational studies. The project was carried out on behalf of the Joint Action ADVANTAGE WP4 group.

**Methods:** The review was performed according to PRISMA guidelines. We searched PubMed, Web of Science, and Embase from 01/01/2002-26/05/2019. Pooled estimates were obtained through random effect models and Mantel-Haenszel weighting. Homogeneity was assessed with the I^2^ statistic. Publication bias was assessed with Egger's and Begg's tests.

**Results:** Of 1027 studies searched, 18 studies were included (*n* = 48,009 participants). Stroke was the only cerebrovascular disease studied in relation to frailty syndromes. All studies except one reported an association between stroke and prefrailty or frailty. However, most studies were not of high quality and there was heterogeneity between results. The pooled prevalence of prefrailty and frailty in stroke patients was 49% (95% CI = 42–57) and 22% (95% CI = 16–27), respectively. The prevalence of frailty was 2-fold in persons with stroke compared to those without stroke (pooled odds ratio = 2.32, 95% CI = 2.11–2.55). Only two studies longitudinally examined the association between stroke and frailty, producing conflicting results.

**Conclusions:** Frailty and prefrailty are common in persons with stroke. These results may have clinical implications, as they identify the need to assess frailty in post-stroke survivors and assess how it may affect prognosis. Better quality, longitudinal research that examines the temporal relationship between stroke and frailty are needed, as well as studies on other types of cerebrovascular disease.

## Introduction

Frailty is a clinical syndrome that is highly prevalent in community-dwelling older adults (1, 2). It is characterized by decreased reserve and function across multiple physiologic systems, leading to a compromised ability to respond to common or acute stressors (3). There is a wide variation of definitions and diagnostic criteria for frailty (4), including those focusing on specific physical factors such as weight loss or slow walking speed (5), as well as more complex definitions that include multidimensional aspects from physical to social, cognitive, and even psychological features (6). In the community, frailty has a prevalence of between 8 and 16% in older adults (1, 2) and is associated with higher risks of adverse outcomes, including death, hospitalization, and disability (3, 5, 7).

A syndrome of prefrailty has also been proposed (sometimes referred to as “intermediate frailty”), which lies on the pathway between being robust and the full frailty syndrome. For example, Fried et al.'s criteria for frailty (5), which are commonly used (4), define frailty according to five criteria: (i) unintentional weight loss; (ii) exhaustion; (iii) low physical activity; (iv) weakness or poor grip strength; and (v) slow walking speed. Frailty is defined as the presence of three or more of these symptoms, prefrailty is defined as fulfilling one or two of the criteria, and robust or non-frail is defined as having none of the five symptoms.

It is becoming increasingly evident that frailty may predispose persons to the development of certain non-communicable diseases, and conversely that chronic disorders may increase the risk of frailty in older individuals (8–14). Such associations have been reported for frailty and chronic kidney disease (14), atrial fibrillation (8), chronic obstructive pulmonary disease (13), anemia (12), and hypertension (9). Further, frailty is associated multimorbidity (10) (the co-occurrence of multiple diseases in a single individual) as well as polypharmacy (11). Both cardio- and cerebrovascular disorders may be associated with frailty. Cardiovascular disease risk scores have been found to predict the incidence of frailty over 10 years in the Whitehall cohort study (15); in particular the Framingham Stroke risk score was associated with a 35% increase in frailty per standard deviation increment. Emerging evidence suggests a link between cerebrovascular disease and frailty; studies report an increased odds of frailty in persons with a history of stroke (16–19), and frailty has been suggested to predict shorter post-stroke survival (20). However, until now no systematic review of the evidence is available to establish what role cerebrovascular disease plays in the development of frailty, and vice versa. The overall objective of the current systematic review and meta-analysis is to examine the relationship between cerebrovascular disease and frailty in adults. The specific aims are: first, to identify the prevalence of frailty in persons with cerebrovascular disease; second, to assess whether frailty is more common in persons with a history of cerebrovascular disease compared to persons without; and third, to examine whether persons with cerebrovascular disease a have a higher risk of developing incident frailty than those without cerebrovascular disease, and vice versa.

## Materials and Methods

### Systematic Review Protocol

The protocol was registered in the international prospective register of systematic reviews PROSPERO (registration number 58303). The review was carried out in accordance with the Preferred Reporting Items for Systematic Reviews and Meta-Analyses (PRISMA) recommendations (21). PICOS was used to define the research question: (i) Population: community-dwelling adults and hospitalized or institutionalized persons aged over 18; (ii) Comparitors: persons with a history of cerebrovascular disease were compared to persons without a history cerebrovascular disease; (iii) Outcomes: frailty defined with an explicit definition/criteria; (iv) Study designs: case-control and cross-sectional and longitudinal cohort studies.

### Search Terms

We searched three databases for relevant articles published from 1st January 2002 to 26th May 2019: (1) Pubmed electronic database of the National Library of Medicine, (2) Web of Science, and (3) Embase. The search was restricted to this time scale in order to focus on studies where a diagnosis of frailty according to standardized criteria were used. Mesh terms and free words referring to frailty and cerebrovascular disease were used as keywords, which were chosen by two physicians. The search terms used in Pubmed was:

(“cerebrovascular disorders”[MeSH Terms] OR “cerebrovascular”[Title/Abstract] OR “stroke”[Title/Abstract] OR “transient ischemic attack”[Title/Abstract] OR “cerebral ischemia”[Title/Abstract] OR “TIA”[Title/Abstract]) AND (“frail elderly”[MeSH Terms] OR “frail^*^”[Title/Abstract] OR “frailty”[Title/Abstract]).

We also screened the reference lists from the selected papers and other relevant articles to identify further relevant studies. The inclusion criteria were: (1) Articles in English or another European language; (2) Study design: cross-sectional, case-control, or cohort studies; (3) Adults only. We excluded letters to the editor, abstracts, conference proceedings, reviews, and editorials.

### Study Selection and Data Extraction

**Figure 1:**
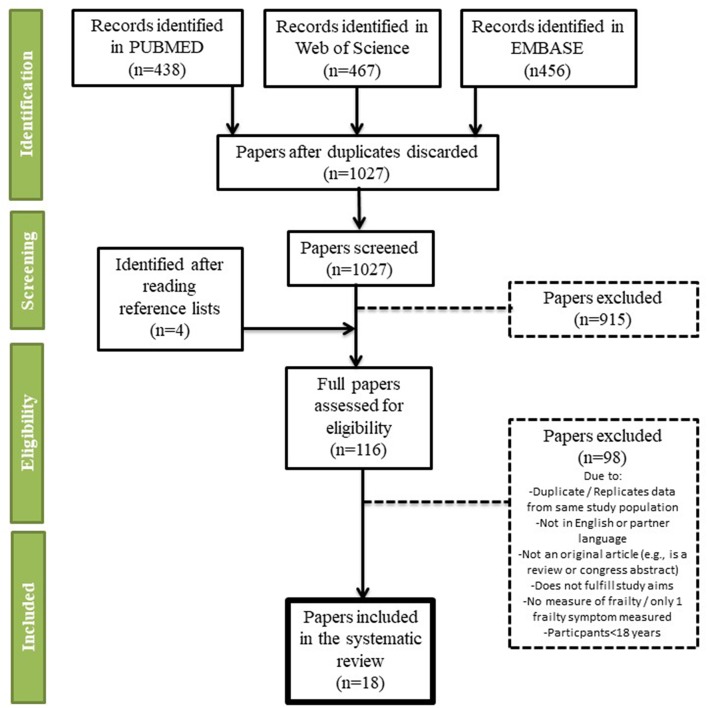
PRISMA flow chart.

The titles and abstracts of the selected studies were independently screened by two assessors. Cross-sectional and longitudinal measures of association between frailty and cerebrovascular disease were selected, as well as case-control studies. Articles were excluded if they (1) did not investigate the aims of the review; (2) included persons younger than 18 years; (3) were not an original article (e.g., editorial, review, or congress abstract); (4) did not provide an explicit definition of frailty; and (5) if frailty was assessed only with a single symptom/measure (e.g., only gait speed or grip strength): (6) was a duplicate; (7) was not in English or another European language; (8) evaluated a composite outcome rather than stroke alone (i.e., included stroke with other neurological diseases or cardiovascular disease). Two assessors read the selected full texts and independently extracted the information from the studies. A third assessor reviewed the data extraction, and any disagreement was resolved through consensus. Articles that were written in another European language than English were sent for translation by a native speaker who conducted the data extraction. The numbers of abstracts screened, and studies assessed for eligibility and included in the review, with reasons for exclusions at each stage, are presented in Figure 1.

Some articles used data from a longitudinal study but the data relevant to our aims were only cross-sectional; in such cases the studies are reported and evaluated as cross-sectional.

### Assessment of Risk of Bias

We evaluated the quality of the studies with the Newcastle Ottawa Scale (NOS) (22). Two assessors independently rated each study, and consensus discussion was used to resolve any disagreement. Score >7 was considered a low risk, 5–7 a moderate risk, and <5 a high risk of bias.

### Statistical Analysis

We performed a meta-analysis on studies that used the same definition of frailty and including only stroke as the measure of cerebrovascular disease. Due to the observational design of the studies, and the methodological differences that may have contributed to a significant share of the variance within the measures of interest, the pooled estimates were obtained through random effect models and Mantel-Haenszel weighting. Homogeneity within the pooled studies was assessed through the I^2^ statistics (significant if ≥ 50%). Publication bias was assessed with the Egger's and the Begg's tests. All statistical analyses were performed with STATA version 14 (statacorp, TX, USA), with *P*-value <0.05 considered statistically significant.

## Results

### General Description of Studies

Of 1,027 papers screened, 116 were selected for full text reading (Figure 1). We included 18 papers for the qualitative and quantitative analysis of the association between cerebrovascular disease and frailty (Table 1), of which one (20) included data only on incident stroke patients with no control population. It should be noted that almost all the studies were designed to examine the association between frailty and a range of chronic diseases, not specifically cerebrovascular alone. Although we included different cerebrovascular diseases in our search criteria, we found no studies on diseases other than stroke. The definition of stroke was very general in all studies, usually self-reported, and only one cross-checked the reports with medical records. None of the studies distinguished between ischemic and hemorrhagic stroke. Studies were mostly from population-based cohorts (15 out of 18), and came from Asia (*n* = 6), Europe (*n* = 5), North America (*n* = 4), and South America (*n* = 2) as well as one that included 8 countries from the 10/66 study. Two studies were conducted only on stroke patients with no control group (16, 26). All studies used Fried et al.'s Cardiovascular Health Study criteria (5) whereas one (31) used a similar five-item frailty scale and one used the Faurot Frailty Index (34). There was a total of 48,009 participants in the 18 studies, and the meta-analysis was based on study populations that used Fried et al.'s Cardiovascular Health Study criteria (5) (*n* = 40,206 participants). All papers that were included in the meta-analysis were from community-based studies.

**Table 1:** Characteristics of the selected studies on cerebrovascular disease and frailty: study methods and main results.

	**Country, study name, population type**	***N***	**Mean age ± SD**	**Women %**	**Cerebrovascular disease diagnosis/definition % prevalence**	Frailty criteria and overall prevalence^**^	**Prevalence % of cerebrovascular disease in each frailty group**	**Odds ratios, risks and other results**	**NOS**
Avila-Funes et al. (23)	France, AMImage study, Community (rural farmers)	176	75 ± 5.2	40	Self-reported history of physician diagnosed stroke. 4.4%	Fried criteria. Overall frail = 18.8%	Robust = 4.0 Frail = 6.3 *p* = 0.630	White matter hyperintensities (mL) higher in frail (mean = 12.1, *SD* = 17.3) than in robust (mean = 4.8, *SD* = 9.6), *p* = 0.23 ADJ: age, sex, education, cardiovascular risk factors. Frail persons had lower Fractional Anisotropy and higher diffusity values in several white matter areas (corpus callosum, anterior limb of internal capsule, external capsule and posterior thalamic radiations).	6
Chen et al. (24)	Japan, Sasaguri Genkimon Study (SGS), Community	1,565	Range 65–93	60	Self-reported history of having ever been diagnosed with stroke. 3.6%	Fried criteria. Overall frail = 9.5, prefrail = 43.9%	Robust = 2.6 Prefrail = 4.2 Frail = 6.0 *p* = 0.02		6
Calado et al. (25)	Brazil, FIBRA study (Study of Frailty in Elderly Brazilian Individuals), Community	385	73.9 ± 6.5	64.7	“Stroke.” Self-report (questionnaire) on any chronic diseases that had been recognized by a doctor during the past year. 2.1%	Fried criteria. Overall frail = 9.1, prefrail = 49.6%	Robust = 0 Prefrail = 2.6 Frail = 8.6 *p* = 0.02		6
de Albuquerque Sousa et al. (26)	Brazil, REDE FIBRA (Network of Studies on the Frailty of Elderly Brazilians), Community	391	74.1 ± 6.6 Range 65–96	61.4	Self-reported presence of stroke diagnosed in the last year. 1.8%	Fried criteria. Overall frail = 17.1%, prefrail = 60.1%	Robust = 1.1 Prefrail = 0.04 Frail = 7.5 *p* = 0.001		5
Espinoza et al. (27)	USA, San Antonio Longitudinal Study of Aging (SALSA), Community	394	Range 65–80	57.6	Stroke was assessed according to self-report of physician-diagnosed disease. 10.7%	Fried criteria. Overall frail = 10.7%		Odds of frailty not significant in multi-adjusted models (data not shown)	7
Lahousse et al. (28)	The Netherlands, Rotterdam study, Community	2,833	Median = 74 Range ≥ 55	55.9	Stroke was “clinically validated.” Prevalence not reported.	Fried criteria. Overall frail = 6%, prefrail = 51%	Robust = 0.8 Prefrail = 0.9 Frail = 2.5 *p* = 0.001		5
Lee et al. (29)	Hong Kong, Community	3,018		49.7	Participants were asked whether they had ever been told by a physician that they had a stroke. Medical diagnoses were cross-checked in the computerized medical system database of the Hong Kong Hospital Authority. Diagnoses were counted as present if reported by the participant or recorded in the medical database. 5.2% in men, 3.5% in women	Fried frailty criteria. 2 year change in frailty status was assessed (e.g., robust worsening (from robust to prefrail or frail), prefrail worsening (from prefrail to frail). Overall baseline frail = 7.9%, prefrail = 50.6%		At 2 year follow-up, about half of prefrail persons remained prefrail, but 11.1% of men and 6.6% of women worsened into frailty, and a quarter recovered into the robust state. Among the frail at baseline, one-quarter remained frail. Change from prefrail to frail in men OR = 1.8 (0.8–3.8), women OR = 2.8 (1.01–7.8). Change from robust to prefrail/frail in men OR = 1.5 (0.7–3.2), women OR = 3.96 (1.4–10.5). In multivariate models stroke was associated with an improvement in men with baseline prefrailty OR = 0.4 (0.2–0.9) or frailty OR = 0.2 (0.1–0.9), and a worsening in prefrail status in women OR = 3.11 (1.05–9.18) and a change from robust to frail/prefrail in women OR = 3.5 (1.2–10.1).	7
Li et al. (30)	China, RulAS population-based survey	1,757	75.3 (3.9)	53.3	Past medical history taken by physicians using a standard questionnaire. 7.3%	Fried criteria, frailty = 10.1% Plus a physical-cognitive frailty scale, frail = 19.4%	Fried Robust = 6.2% Frail = 17.8% *p* = 0.001 Physical-cognitive frailty Robust = 5.9% Frail = 13.6% *p* = 0.001		7
Llibre Rodriguez et al. (16)	8 countries, 10/66 Study, Community	1,6886	≥65	62.4%	Stroke was self-reported, but confirmed by the interviewer as having characteristic symptoms lasting for more than 24 h. 6.7%	Modified Fried criteria (only four indicators measured)		Pooled estimates (10 sites in 8 countries) Stroke and frailty OR = 2.3 (2.1–2.6) Adj: age, sex, education	7
Merchant et al. (31)	Singapore, HOPE (Healthy Older People Everyday), Community	1,051	71.2	57.2	Patients screened for chronic diseases, including stroke	5-Item frailty scale (fatigue, resistance, ambulation, illness, loss of weight) (32) Overall frail = 6.2%, prefrail = 37%	Robust = 1.7 Prefrail = 6.4 Frail = 16.9 *p* < 0.001		5
Nadruz et al. (33)	USA, Atherosclerosis Risk in Communities Study, Community	3,991	75.6 ± 5.0	59	Previous Stroke. 2.7%	Fried criteria. Overall frail = 5.3%	Robust = 2 Frail = 7 *p* < 0.001		6
Ng et al. (18)	Singapore, SLAS—Singapore Longitudinal Aging Studies I and II, Community	1,685	66.7 ± 7.76	64	The self-report of a medical disorder diagnosed and treated by a physician(s) was recorded for 22 named diagnoses, including stroke. 32%	Fried criteria. Overall frail = 5%, prefrail = 42%	Robust = 1.6 Prefrail = 4.1 Frail = 12.1 *p* < 0.001	Significant correlates of prefrailty-frailty from binary logistic regression via backward stepwise variable selection: Stroke B = 0.76 OR = 2.1 (1.1–4.1), *p* = 0.23	5
Seamon et al. (34)	USA, Medicare sample	7,258	79.4 (8.4)	56.7	All patients hospitalized with a first-time acute ischemic stroke. 100%	Faurot Frailty Index		39.1% of stroke patients were robust, 36.0% were prefrail, and 24.9% were frail.	4
Serra-Prat et al. (17)	Spain, Community	154	80.1 (3.5)	47.5	Information on comorbidities and medications was obtained from the electronic medical records held by the corresponding centers. All other information was obtained directly from the patient by trained healthcare professionals. Prevalence not reported.	Fried criteria. Overall frail = 53.7%, prefrail = 14.2%	Robust = 8.7 Prefrail = 7 Frail = 23.9 *p* = 0.003	Crude OR for frailty in stroke patients = 3.82 (1.7–8.58). Adjusted OR = 4.5 (1.35–14.97), *p* = 0.014.Adj: age, sex, education, anorexia, osteoarthritis, dyspepsia, number of medications, anemia, CRP, muscle mass, and creatinine.	6
Taylor-Rowan et al. (35)	UK, Patients consecutively admitted to acute stroke unit	545	69 (14)	46	Physician diagnosed. 100%	33 item frailty index.		28% of stroke patients were frail and 51% were prefrail.	4
Trevisan et al. (36)	Italy, Progetto Veneto Anziani, Community	2,925	74.4 ± 7.3 Range ≥ 56	59.7	Personal interview, medical interview and clinical examination including blood tests. Cardiovascular disease (CVD) was defined as atrial fibrillation; congestive heart failure; angina pectoris requiring a stent, angioplasty, or hospitalization; myocardial infarction; or stroke. Prevalence not reported.	Fried criteria. Overall frail = 6.6%, prefrail = 49.3%		Of the persons who were nonfrail at baseline 26.7% became prefrail at follow-up and 6.3% became frail. Progressing from prefrail to frail stroke OR = 1.96 (1.72–2.24), *p* < 0.001	8
Vaingankar et al. (19)	Singapore, Well-being of the Singapore Elderly study, Community	2,102	69 Range ≥60		Not specified. Field interviewers collected data on medical conditions. “Stroke”	Fried criteria. Overall frail = 40.1%, prefrail = 5.7%	Robust = 2.2 Prefrail = 7.4 Frail = 13.4 *p* < 0.001	Prefrailty OR = 2.6 (1.2–5.8), *p* = 0.018; Frailty OR not significant (data not shown)	6
Winovich et al. (20)	USA, Cardiovascular Health Study, subsample of persons with incident ischemic stroke	893	82 ± 6.4	61	Incident stroke events identified through semi-annual phone calls, hospital discharge report review, and health insurance registries.	Fried criteria.		In patients with incident stroke, 27.8% were robust, 54.9% prefrail and 17.3% frail. No comparison between no stroke controls.	5

*ADJ, adjustment variables; ADL, activities of daily living; CMB, Cerebral Microbleeds; CRP, C-reactive protein; CT, Computed tomography; CVD, cardiovascular disease; DSM-IV, Diagnostic and Statistical Manual of Mental Disorders, 4th Edition; HR, hazard ratio; MRI, magnetic resonance imaging; NOS, Newcastle Ottawa Scale risk of bias score; OR, odds ratio; VaD, vascular dementia*.

* *All numbers are rounded to one decimal point*.

** *In all studies using Fried criteria, the cutoff for frailty was 3 out of 5 symptoms, and prefrailty was 1–2 symptoms*.

### Prevalence of Prefrailty and Frailty in Persons With Stroke

**Figure 2:**
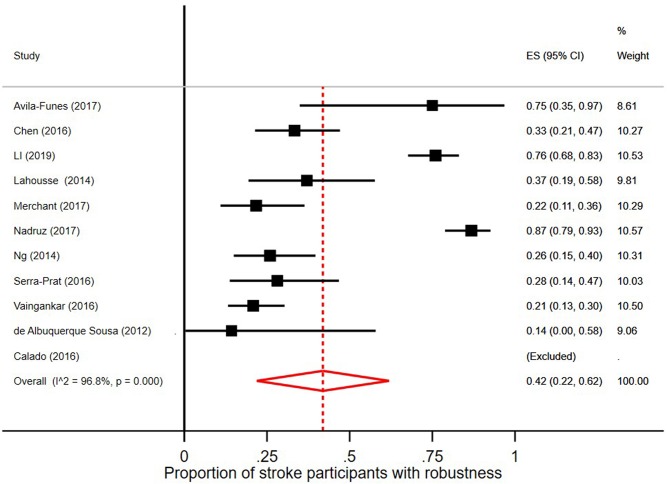
Proportion of stroke participants who were robust/without frailty [according to Fried et al. (5) criteria].

**Figure 3:**
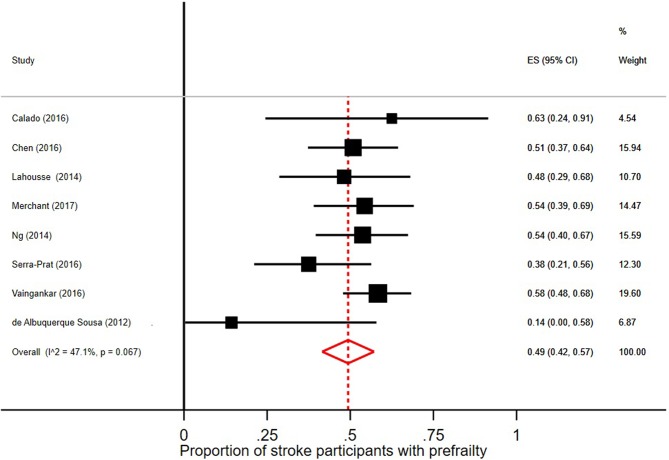
Proportion of stroke participants with prefrailty [according to Fried et al. (5) criteria].

**Figure 4:**
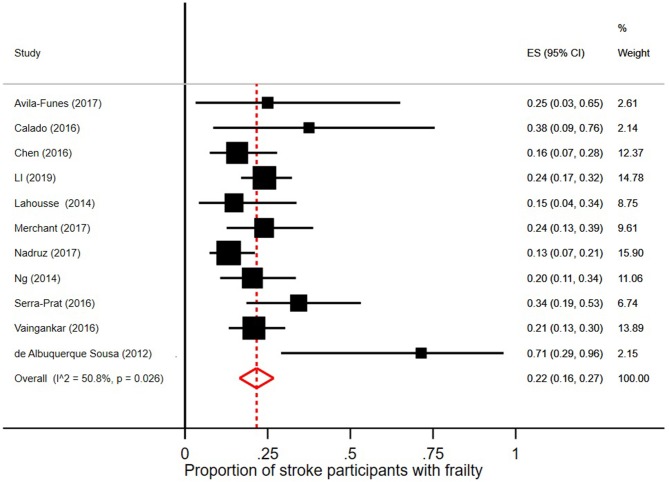
Proportion of stroke participants with frailty [according to Fried et al. (5) criteria].

In the 18 studies selected, the percentage of frail persons with stroke ranged between 2.5 and 24.0%. Studies were included in the meta-analysis if they defined frailty according to Fried et al.'s Cardiovascular Health Study criteria (5) and had stroke as independent variable. The pooled prevalence figures showed that 42% of stroke patients were not frail or prefrail, classified as robust (95% CI = 22–62; *I*^2^ = 96.8%), see Figure 2. The pooled prevalence of prefrailty in stroke patients was 49% (95% CI = 42–57; *I*^2^ = 47.1%), see Figure 3, and the pooled prevalence of frailty in stroke patients was 22% (95% CI = 16–27; *I*^2^ = 50.8%) as shown in Figure 4.

**Figure 5:**
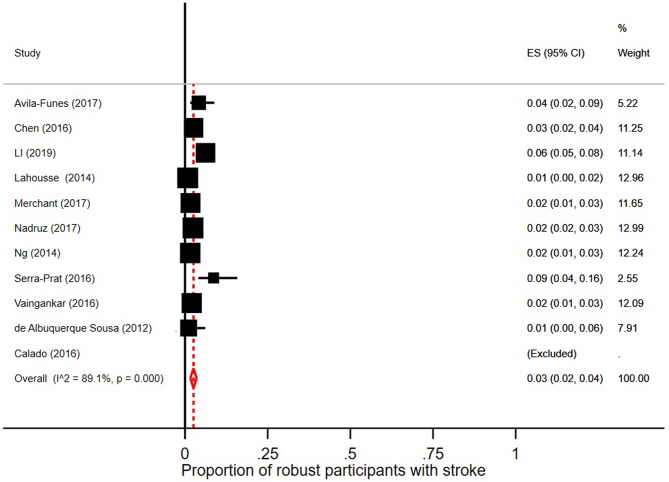
Proportion of robust participants [according to Fried et al. (5) criteria] with stroke.

**Figure 6:**
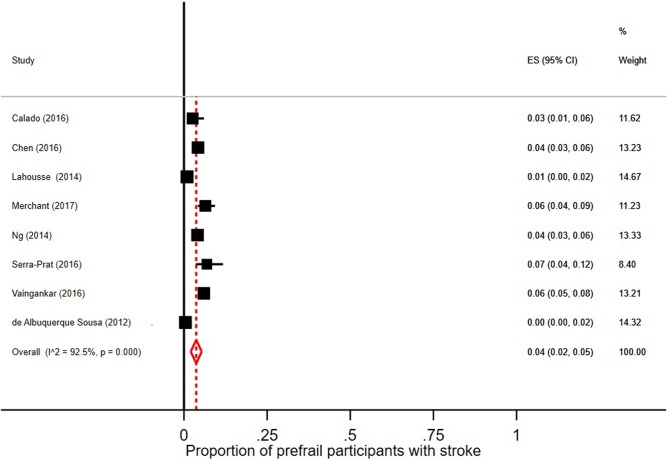
Proportion of prefrail participants [according to Fried et al. (5) criteria] with stroke.

**Figure 7:**
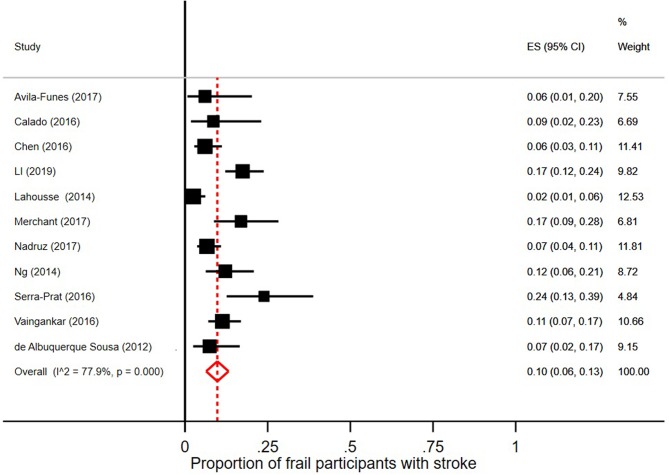
Proportion of frail participants [according to Fried et al. (5) criteria] with stroke.

Few robust individuals had a diagnosis of stroke, see Figure 5, with a pooled prevalence of 3% (95% CI = 2–6; *I*^2^ = 89.1%). The pooled prevalence of stroke in prefrail individuals was 4% (95% CI = 2–5; *I*^2^ = 92.5%) as shown in Figure 6. The pooled prevalence of stroke in frail individuals was 10% (95% CI = 6–13; *I*^2^ = 77.9%), see Figure 7.

### Association Between Prefrailty, Frailty, and Stroke

**Figure 8:**
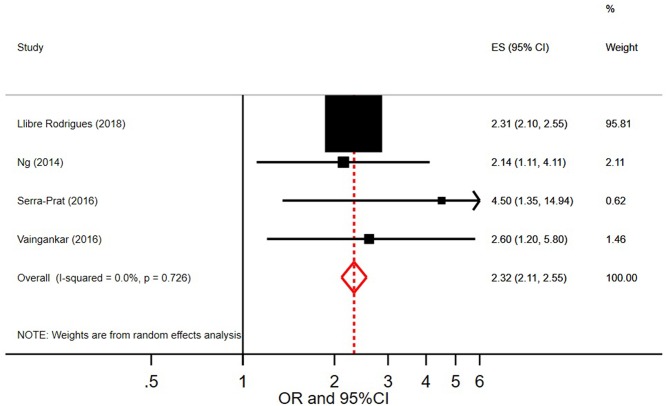
Pooled odds ratio and 95% Confidence Intervals on the association between stroke and frailty.

Of the nine studies that measured bivariate associations, all reported a significant association between frailty and stroke except one (23). Figure 8 shows the pooled odds ratio of stroke and frailty. The study by Llibre Rodriguez et al. (16) includes a pooled OR using data from 10 sites in the 10/66 study (from Cuba, Dominican Republic, Puerto Rico, Peru, Venezuela, Mexico, and India). When pooling this OR with those from the other 4 studies identified in our review, we found that persons with stroke were more than twice as likely to be frail than persons without stroke (pooled OR = 2.32, 95% CI = 2.11–2.55; *I*^2^ = 0.0%).

### Longitudinal Associations Between Stroke and Frailty

Only two studies longitudinally analyzed the change in frailty status in individuals over time. Trevisan et al. (36) found that stroke was only associated with a transition from prefrail to frail. Lee et al. (29), however, found that the associations differed according to sex; in multivariate models stroke was associated with an improvement in men with baseline prefrailty or frailty, but in women it was associated with a worsening of frailty status, both in terms of transitioning from robust to prefrail/frail, and from prefrail to frail.

### Assessment of Bias

The quality of studies differed, with only some conducting extensive multivariate analyses to account for potential confounders. In the tables, we report these study results in detail to highlight how any associations between frailty and cerebrovascular disease changed in significance between crude and adjusted analyses. The NOS scores are show in Table 1. The majority of studies (*n* = 15) had a moderate risk, one had a low risk, and two had a high risk of bias. The main reasons for bias were samples only on one sex, and selection bias or missing data. Many of the studies only used self-report as a measure of cerebrovascular disease. The two studies without stroke-free control groups were scored as having a high risk of bias. There was evidence of publication bias in our meta-analyses according to the Egger's test (*p* = 0.022) although the Begg's test was not significant (*p* = 0.602).

## Discussion

### Summary of Main Findings

This systematic review highlighted that, although almost all studies on the topic demonstrate an association between stroke and frailty, there is high heterogeneity and the quality of studies is generally only moderate. The results of the meta-analysis should be taken with caution, but they suggest that a substantial proportion of stroke patients have either frailty or prefrailty, with a pooled prevalence of 21 and 48%, respectively. Due to the current low prevalence of stroke survivors in community studies, the prevalence of stroke in persons with frailty was quite low; 10%. Persons with a history of stroke were more than twice as likely to be classified as frail than those without stroke. Finally, this review identified a lack of longitudinal studies examining the temporal relationship between frailty and stroke, as there were only two such papers.

### Interpretation

There are several explanations for the association between cerebrovascular disease and frailty.

Certain specific symptoms of frailty such as reduced walking speed are likely to be directly related to the physical disability that patients experience after a cerebrovascular event. From this perspective it may be relevant to discuss whether the criteria for frailty changes across disease states. It is worth noting, however, that although some of the symptoms may be directly related to cerebrovascular disease itself, the two conditions are not synonymous; indeed our meta-analyses showed that a substantial proportion of people with a history of a stroke were robust [i.e., did not exhibit any of the five criteria for frailty proposed by Fried et al. (5)]. In addition, frailty diagnosis in persons with illness can provide important clinical prognostic information; regardless of which frailty scale is used to diagnose frailty within older patients admitted to hospital, severity of frailty is predictive of poor discharge outcomes such as death, poor quality of life, need for community, and hospital readmittance (37). Further, several studies that have identified vascular alterations in persons with frailty. In a community-based study in Taiwan (38), only 11.1% of robust persons had a cerebral microbleed compared to 17.9% of prefrail and 34.4% of frail persons. Cerebral microbleeds specifically in the brainstem were associated with a 13-fold increased odds of frailty. The study by Avila-Funes et al. (23) that was included in the current review also provided imaging data, showing that white matter hyperintensities were higher in frail than in robust persons, similar to an Australian study (39). Further, frail persons had lower Fractional Anisotropy and higher diffusity values in several white matter areas (corpus callosum, anterior limb of internal capsule, external capsule and posterior thalamic radiations). A Korean study also demonstrated the association between retinal microvascular changes and/or white matter hyperintensities and frailty in older persons (40). Therefore, it may be that frailty and vascular changes are already associated before the onset of an overt cerebrovascular event.

Interestingly, a French longitudinal study (41) reported that both frailty and prefrailty increased the risk of incident vascular dementia over 7 years. Frailty may also be related to cerebrovascular disease via related factors, such as hypertension.

It is important to consider the effect of post-stroke survival when interpreting the results of this review. Estimates concerning frailty in persons with a history of cerebrovascular disease will change depending on the survival rates following cerebrovascular incidents in different study locations. As survival rates increase, it is likely that frailty incidence subsequently increases in person who have had a non-fatal stroke. Frailty also poses challenges in the treatment of acute illness. For example, a recent study (42) examined the way that concurrent frailty affects persons presenting for hospital care with acute illnesses. They found that the severity of the acute illness was directly associated with mortality risk in persons with severe frailty. Indeed, it is worth noting that 42% of persons with a history of stroke were classified as robust. This suggests that frailty is not an inevitable outcome of cerebrovascular disease, although with the current data we cannot determine how long since stroke onset frailty was measured. Moreover, this may demonstrate a selection bias, where persons included in studies represented those with milder cases of stroke that did not result in mortality. Nevertheless, the figures also provide a positive outlook that stroke may not necessarily be associated with frailty or prefrailty in the long-term. More research into the temporal relationship and changes over time in frailty status in stroke patients are needed.

During the abstract screening we also identified two studies that did not look specifically at cerebrovascular disease but cardiovascular diseases as a whole (43, 44). The first Italian study provided important results concerning the association between prefrailty and a composite of cardiovascular disease (including coronary disease, heart failure, and cerebrovascular disease). This community study followed persons free from baseline frailty over 4.4 years. The age-adjusted incidence of cardiovascular disease was 75 events per 1,000 person-years, but only 8 of the 84 events were stroke. The risk of incident cardiovascular disease was 30% higher in persons fulfilling one frailty criterion at baseline, and 80% higher for those with two criteria. In the second study, also from Italy (44), frailty increased the risk of cardiovascular disease (revascularization, myocardial infarction, stroke, or heart failure) by 35% over 8.7 years, with a stronger association seen in women. These studies provide important information relating to cerebrovascular disease but unfortunately no separate results for the specific diseases were presented. We acknowledge that there may be other studies that include stroke within a composite measure of cardiovascular disease and highlight that out review was not aimed to identify or include such studies.

According to our meta-analysis, one in five participants with stroke were also frail but it is noteworthy that the remaining persons were not all robust; half of them were classified as prefrail. Prefrailty (sometimes referred to as “intermediate frailty”) is also a relevant clinical condition associated with an increased risk of adverse outcomes. Frailty is a dynamic and progressive condition, and prefrailty may represent the intermediate stage between healthy aging and the predisposition to catastrophic events (5), where physiologic systems have already started to decline but individuals still preserve a certain level of resilience to stressors or adverse outcomes. It is important to highlight that prefrail individuals may have the possibility to improve, whereas <1% of frail persons return to a robust status over time (45). Preventative strategies at this stage of prefrailty may, therefore, be appropriate in terms of disease management (for example post-stroke care and treatment) and prevention of negative outcomes. Indeed a physical training intervention was shown to be beneficial for preventing functional decline in prefrail older persons but not those with severe frailty (46). Thus, as discussed in more detail below, care systems that examine the occurrence of prefrailty or frailty in chronic diseases such as stroke may help identify groups that may need specific intervention strategies or tailored care needs (47, 48).

### Limitations and Strengths

There were several limitations that should be considered when interpreting the results of this systematic review. First, the primary aim of many of the studies was not to specifically investigate cerebrovascular disease; they were mostly studies examining multiple chronic diseases in relation to frailty. Second, the majority of studies measured history of stroke by self-report only, which may lead to measurement bias. None of them distinguished between ischemic and hemorrhagic stroke, and no studies reported any other forms of cerebrovascular disease. Our search criteria did not specify less common cerebrovascular diseases such as leukoaraiosis or chronic subcortical vascular encephalopathy, but the key word “cerebrovascular disease” did not identify any papers on these diseases. Most studies measured only history of stroke, though it would be interesting to differentiate between recent cerebrovascular disease with events occurring many years before. In addition, despite considerable overlap between cerebrovascular and cardiovascular disorders, few studies in the literature are available that examine these conditions together, or account for the presence of the other and its effect on frailty. Therefore, our review focused only on cerebrovascular disease. An important limitation is that there was great heterogeneity in the meta-analysis, possibly due to the lack of high-quality studies. Therefore, the results of the meta-analyses should be taken with caution. However, it is worth noting that all studies expect one reported the same direction of results, i.e., an association between frailty and stroke. Further, there were only two studies investigating the longitudinal association between frailty and stroke. Causal associations were, therefore, impossible to assess. Although many studies adjusted for confounders there is potential for residual confounding that may affect the relationship between frailty and stroke. For example, a recent systematic review reported that both prefrailty and frailty are associated with polypharmacy (11) and some stroke patients may be at risk of having multiple medications, especially older individuals. Yet few studies controlled for this. Likewise, the issue of anticoagulants treatments or similar agents has not been explored – possibly because most of the studies were not designed with the primary aim to investigate the association between stroke and frailty but looked at a variety of diseases. Another important issue is that it is difficult to compare study results due to the wide variation of frailty indices. Dent et al. (4) discuss the wide range of tools used to measure frailty and the differences between them. However, it is worth noting that for the majority of studies Fried et al.'s criteria were applied (5). Unfortunately, most of the studies did not provide sex-stratified figures for the association between frailty and stroke, and therefore, we were unable to investigate this issue. Indeed, results from Lee et al. (29) suggest that there may be sex-differences in the association between stroke and frailty; they reported that stroke was associated with worsening frailty status in women whereas the opposite pattern was seen for men. Several strengths of the review should also be noted. First, we included an extensive literature search with three medical databases, and all abstract screening and data extraction was conducted independently by two researchers. Further, the inclusion of articles in any European language is a major strength.

### Relevance

Currently the evidence is not clear enough to make any clinical or health policy recommendations concerning frailty and stroke based on the results of this systematic review as there was heterogeneity between studies and few were high quality with low risk of bias. Additional studies, particularly longitudinal ones, are needed before any information should be used in clinical settings. Clinically it may be important for clinicians to communicate the potential risk of frailty in relevant patients, to possibly include frailty assessment in follow-up visits, and to consider frailty in any care planning. Increasing opinion is highlighting the need for integrated and multidisciplinary care models that take into account comorbid conditions, geriatric syndromes such as frailty, and clinical as well as non-clinical needs (47, 48). It is also becoming evident that frailty needs to be taken into account when treating chronic diseases (49), particularly hypertension, which has important clinical implications for cerebrovascular disease. Blood pressure control is an essential aspect of post-stroke treatment and prevention of additional cerebrovascular events, but at the same time one report suggested that antihypertensive treatment might increase the risk of frailty by 77% (15). It is a priority to untangle such a relationship and investigate the proper blood pressure threshold that guarantees an adequate care of post-stroke patients but that at the same time prevents frailty. We also need future research to ascertain whether frailty assessment affects response to rehabilitation or whether the outcomes of post-stroke rehabilitation differs according to whether the patient is also frail (50, 51). Studies have also suggested that pre-stroke frailty is associated with lower post-stroke cognition (52), independently from factors that have been previously associated with post-stroke cognitive impairment. Therefore, frailty may play an important role not just in physical but also cognitive recovery.

### Future Research

Several avenues for future research have been identified after conducting this review. The most crucial is the need for longitudinal studies that look clearly at how changes over time before, during, and after the onset of a stroke, in order to determine whether frailty affects the onset of stroke, or whether stroke causes frailty. Of particular interest are the specific transitions from robust to prefrail and frail, progression from prefrailty to frailty, and how a person can decline or improve in status over time. It is noteworthy that the majority of studies applied Fried et al.'s criteria (5) and few used more complex definitions such as those by Rockwood et al. (6) that are gaining increasing interest in both clinical and research settings. Future studies comparing different frailty indices may produce more informative results concerning how stroke relates to different frailty types and symptoms. Further, the need for more large studies, specifically aimed at assessing cerebrovascular disease, are needed, with clinically validated measures (not just self-report), which look at any variations according to the different types of cerebrovascular disease, and that take into account clinical factors such as time since disease onset, response to therapy, and other aspects that may affect frailty status. In addition, there is a need for studies that examine both cardio- and cerebrovascular conditions together, which carefully examine the combined and individual effect of the two disorders in relation to frailty. Some studies also noted differences between men and women, suggesting a possible stronger association between stroke and frailty in women than men (29). Therefore, more research stratifying for sex and adjusting for multiple potential confounders is needed. Age might also affect the association between stroke and frailty and although most of the studies in the current review adjusted their odds ratios for age, the pooled prevalence analyses were unadjusted and some studies included much older patients than others. Finally, an interesting avenue for future research is what affect frailty has on the treatment of cerebrovascular disease.

## Conclusions

In conclusion, the current review indicates that there may be an association between stroke and frailty or prefrailty, but no causal association between the two conditions can be established with the current evidence. As stroke survival rates increase, it may be likely that frailty incidence subsequently increases in person who have had a non-fatal stroke.

## Author Contributions

KP and DV contributed equally to the study and were responsible for the study design, abstract screening, data extraction, performed the meta-analysis, interpretation of results, and writing of the manuscript. GO and RB were responsible for the study design, interpretation of results, and critical revision of the manuscript. LP, VR, CR, BS, and AM conducted the abstract screening, data extraction, and critically revised the manuscript.

### Conflict of Interest

The authors declare that the research was conducted in the absence of any commercial or financial relationships that could be construed as a potential conflict of interest.
